# Non-Carcinogenic Health Risk Assessment due to Fluoride Exposure from Tea Consumption in Iran Using Monte Carlo Simulation

**DOI:** 10.3390/ijerph16214261

**Published:** 2019-11-02

**Authors:** Mohammad Amin Karami, Yadollah Fakhri, Shahabaldin Rezania, Abdol Azim Alinejad, Ali Akbar Mohammadi, Mahmood Yousefi, Mansour Ghaderpoori, Mohammad Hossien Saghi, Mohammad Ahmadpour

**Affiliations:** 1 Department of Environmental Health Engineering, School of Health and Nutrition, Lorestan University of Medical Sciences, Khorramabad, Iran; karami.mohammadamin@yahoo.com; 2 Department of Environmental Health Engineering, Student Research Committee, School of Public Health and Safety, Shahid Beheshti University of Medical Sciences, Tehran, Iran; Ya.fakhri@gmail.com; 3 Department of Environment & Energy, Sejong University, Seoul 05006, Korea; 4 Department of Public Health, Fasa University of Medical Sciences, Fasa, Iran; azimalinejad@gmail.com; 5 Department of Environmental Health, Neyshabur University of Medical Sciences, Neyshabur, Iran; 6 Department of Environmental Health Engineering, School of Public Health, Iran University of Medical Sciences, Tehran, Iran; mahmood_yousefi70@yahoo.com; 7 Nutrition Health Research Center, Lorestan University of Medical Sciences, Khorramabad, Iran; 8 Environmental Health Engineering, Non-Communicable Diseases Research Center, Sabzevar University of Medical Sciences, Sabzevar, Iran; saghi9@gmail.com; 9 Health Education and Promotion, Department of Public Health, Maragheh University of Medical Sciences, Maragheh, Iran; ahmadpour.mohamad8@gmail.com

**Keywords:** Fluoride, Tea, Iran, Health risk assessment, Monte Carlo analysis

## Abstract

Excessive intake of fluoride can cause adverse health effects. Consumption of tea as a popular drink could be a potential source of fluoride exposure to humans. This research aimed to evaluate the fluoride concentration in tea among the Iranian people using the available data in the literature and to assess the health risk related to the consumption of tea in men, women, and children. The health risk assessment was conducted using the chronic daily intake and hazard quotient according to the approach suggested by the Environmental Protection Agency. The fluoride content in published studies varied noticeably, ranging from 0.13 to 3.27 mg/L. The results revealed that the hazard quotient (HQ) in age groups of women (21–72 years) and children (0–11 years) was within the safe zone (HQ < 1) which showed that there was no potential of non-carcinogenic risk associated with drinking tea in these groups. However, in one case of the men (21–72 years), the HQ > 1 which shows a probable risk of fluorosis. The order of non-carcinogenic health risks in the studied groups was in the order of men > women > children. The results of this can be useful for organizations with the responsibility of human health promotion.

## 1. Introduction

Fluoride, as one of the main trace elements, is extensively found in nature. The most common form of fluoride is the ionic form that has a high electronegativity. This property gives the capability to react and bind to various compounds [1,2]. In the human body, fluoride is absorbed from the gastrointestinal tract. Besides, fluoride in appropriate concentration has a vital role in the maintenance of teeth and bone health. However, a high concentration of fluoride has adverse effects on human health [3]. According to the World Health Organization (WHO), fluoride at levels of 0.5–1.5 mg/L plays a vital role in preventing adverse effects in drinking water [4]. For instance, a fluoride concentration of less than 0.5 mg/L leads to dental cavities [5]. Based on a previous study, regular consumption of water containing at least 0.9 mg/L of fluoride accounts for at least 37 percent of dental fluorosis cases [6]. Concentrations higher than 1.5 mg/L can lead to several health hazards such as hypertension, nervous system damage, infertility, thyroid, urinary tract disease, and dental and skeletal fluorosis in children and adults [7]. A part of the adsorbed fluoride can accumulate in the bone tissues because of its tendency for calcium and magnesium. Adsorbed fluoride mostly accumulates in the teeth [8].

Drinking water is the most important source of human exposure to fluoride [9]. It is estimated that approximately more than 200 million people living in 20 developing and developed countries receive fluoride in drinking water at a higher concentration than the WHO levels [6,7,10]. Although drinking water is the main source of fluoride intake in humans, other sources such as breathing, skin contact, and food uptake can also be potential routes of fluoride exposure to the human body [4]. Therefore, tea can also be a possible source of fluoride for humans. The tea plant is considered as a fluoride accumulator that contain high levels of fluoride depending on the growth conditions such as acidity and calcium content of the soil [11]. The result of previous studies showed that the accumulation of fluoride in tea plants is proportional to the concentration of fluoride in the soil [12]. Generally, the total fluoride concentration in tea leaves can vary depending on the variety and location [10,11,13,14]. According to Yi, J and Cao, J. studies, fluoride content in tea leaf can vary from 2.1 to 1175 mg/kg, 0.49 to 631 mg/L for instant tea and 0 to 33.4 mg/L for the tea beverage [15]. In addition, Malinowska et al. reported fluoride levels of 0.02–0.09 mg/L in herbal tea infusion [16]. Compared to young leaves, the fluoride content is 10 to 20 times higher in old leaves and shoots [17].

Tea is one of the most important non-alcoholic beverages in the world and has received considerable attention as an important health drink. Its consumption has increased significantly worldwide in the last decades. Drinking tea as a health drink has many benefits such as antioxidant, antimutagenic, and anti-carcinogenic activities as well as preventive effects against cardiovascular diseases, diabetes, and obesity [3,16,18]. The levels of exposure to fluoride in tea can be influenced by infusion time, brand, consumption frequency and the quantity of used tea [11]. High concentrations of fluoride in tea leaves have been reported from teas in different parts of the world such as Iran [3] Poland [16], China [19], and Turkey [20].

Kakumanu and Rao (2013) reported a case of skeletal fluorosis due to the consumption of tea. The patient was a 47 year old woman who usually consumed a pitcher of tea made from 100 to 150 tea bags daily (estimated fluoride intake, >20 mg per day). Based on the patient’s medical records, the physicians concluded that consumption of tea was the main reason for the skeletal disorder [10].

In Iran, there is a strong desire for tea drinking, and it is an important part of Iranian diets. It is estimated that consumption of black tea is about 1.5 kg per capita in Iran and about 4.5 percent of the whole consumed tea in the world [21]. On average, one liter of tea is consumed by a person per day in Iran [22]. Results of previous studies showed that the concentration of fluoride in most popular brands on tea infusion in Iran varied from 0.13 to 3.27 mg/L [3,17,23]. Therefore, tea can be one of the most important fluoride exposure sources in Iranian people. Given the potential adverse effects from exposure to fluoride, this research was conducted to assess the health risks associated with the consumption of tea among Iranian people.

### The Purpose of this Study

The aim of this study was the investigation of fluoride concentrations in consumed tea for the Iranian population. Also, the non-carcinogenic risks due to exposure to fluoride through tea to consumers (men, women, and children) using the available data in previous studies were assessed. In this study, the U.S. Environmental Protection Agency (USEPA) risk assessment model and Monte-Carlo simulation technique were applied.

## 2. Materials and Methods

### 2.1. Study Area Description

Iran, an ancient country located in the Middle East, a region between Asia, Europe, and Africa with a surface area of 1,648,195 km^2^ is the second-largest country in this region and the 18th-largest in the world [24]. It has almost 81 million population and ranks as 19th in the world [25]. It is located at latitude 32.42° N and longitude 53.68° E. Because of the geographical location and climate variations, Iran has a rich ecology. Most of the Iranian area (65 percent of the country) is arid, 20 percent of the country is considered semi-arid, and the rest has a humid or semi-humid climate. High variation in temperature from −20 to +50 °C can be seen during the year. The yearly precipitation varies highly, ranging from less than 50 mm to about 1000 mm. The greatest rainfall occurs in the western part of the Caspian, where it reaches beyond 1000 mm and sometimes to 1900 mm. The average annual precipitation is 250 mm which is less than one-third of the average annual precipitation in the world. The uneven distribution of precipitation causes some of the land areas to have severe drought. Generally, Iran has an arid climate. Today, the water crisis is an issue that confronts Iranians with security challenges.

### 2.2. Data Collection and Analysis

In the present study, the concentrations of fluoride in different brands of consumed tea by Iranian people were reviewed from research published articles from 2006 to 2018. The articles were obtained from international and Iranian databases such as science direct, google scholar, the web of science, Scopus, PubMed, magiran, Irandoc, scientific information database, and the information institute for science. After the initial search, the articles were monitored for their eligibility to be included in this research. Then, the important information was extracted for more analysis.

**Figure 1:**
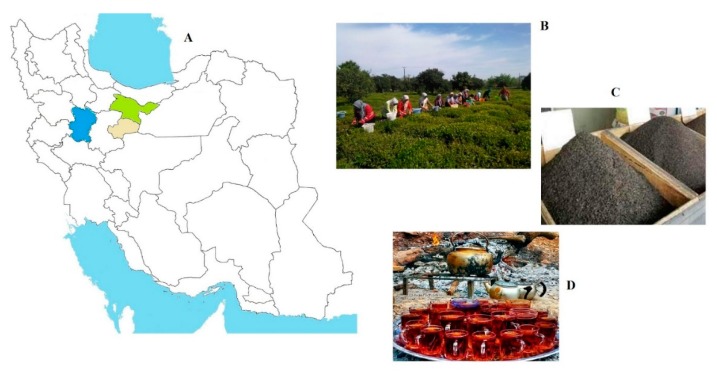
Location map of performed studies related to fluoride in consumed tea in Iran. (**A**): Distribution Studies, (**B**): Harvesting tea, (**C**): Dried and processed tea, (**D**): Tea made for drinking.

During the analysis, the used materials were extracted from reports, cross-sectional studies, and similar published studies in the field. The extracted information of each study consisted of tea type, brand type, sample number, time (year), and concentration (mean, minimum, and maximum). Finally, five studies were selected as shown in Table 1. The general information such as study area, tea type, fluoride concentration as well as standard deviation (SD) was summarized. Also, the location map of the studies is shown in Figure 1.

### 2.3. Health Risk Assessment

Human-health risk assessment is the process of evaluating the probability of harmful effects on human health on exposure to specified chemical agents for a certain period. The health risk assessment can be classified in terms of carcinogenic and non-carcinogenic health risks and generally is based on the determination of risk level [29,30,31,32]. There are several routes through which humans can be exposed to contaminants. These routes include dermal, inhalation, and ingestion pathways. In this study, risks from ingested fluoride through tea consumption were evaluated. The target population was categorized as follows: men and women (21–72 years) and children (0–11 years). The results of fluoride analysis in black tea were used to determine the lifetime chronic daily intake (CDI) as mg/kg/day associated with this element. Based on the US EPA recommendations, the chronic daily intake was estimated using Equation (1) [33].

(1) $$\mathrm{CDI}=\frac{C.\mathrm{IR}.\mathrm{EF}.\mathrm{ED}}{\mathrm{BW}.\mathrm{AT}}$$

where CDI and C are the chronic daily intake (mg/kg/day) and the mean concentration of target chemical (mg/L), respectively.

Ingestion rate (IR) of water (L/day) and Exposure frequency EF (d/year) are the water respectively.

Also: exposure duration (ED) for cancer risk assessment (year), the average body weight (BW) (kg) for the age groups and averaging time AT (day) = (ED × 365) respectively. 

The used parameters for the calculation of the CDI values are given in Table 2. These parameters were selected from previously published studies. The Hazard Quotient (HQ) was determined by dividing the CDI by the standard value for non-carcinogenic impacts using the reference dose (RfD) considered by the USEPA. For assessment of health risk related to fluoride exposure, HQ was used in different age groups in men and women (21–72 years), and children (0–11 years) in Iran. The HQ of the non-carcinogenic risk assessment for fluoride exposure through tea consumption was calculated using Equation (2).

(2) $$\mathrm{HQ}=\frac{\mathrm{CDI}}{\mathrm{RfD}}$$

In this study, oral RfD was 0.06 mg/kg/day [34]. With HQ value less than one, adverse health effects are unlikely to develop for exposed people. If the value of HQ is higher than one, there is a chance that non-carcinogenic impacts may occur.

### 2.4. Monte Carlo Simulation

Health risk assessment is a procedure based on the deterministic levels for input data using the chemical concentration and other risk model parameters. Evaluating the average or maximum level by the risk assessment model, sometimes underestimates or overestimates the actual risk related to the low occurrence possibility [34]. Considering that human health is associated with some uncertainty, ignoring these uncertainties can lead to the loss of information and, therefore, mistakes can be made and unrealistic decisions associated with human health protection taken [35]. Based on the literature, Monte Carlo simulation as one of the probabilistic approaches has been applied in order to assess realistic risk related to chemical substances. This approach has the capability to minimize uncertainty. In the Monte Carlo simulation method, random values are repeatedly selected from the probability distribution of several inputs to obtain the probability distribution of risk [36]. In the Monte Carlo simulation, instead of using a single-point value, different variable values are used, and the calculation is repeated frequently, and finally, the results can be obtained with different levels of assurance between one percent and 99 percent. The probabilistic approaches have been applied widely to examine the potential harmful hazards of pollutants in water and other media. The Oracle Crystal Ball software (version 11.1.2.4.600, Build 11.1.4512.0 on 1/11/2016) was used to perform the Monte Carlo simulation calculations.

## 3. Results and Discussion

### 3.1. The Concentration of Fluoride in Tea

**Figure 2:**
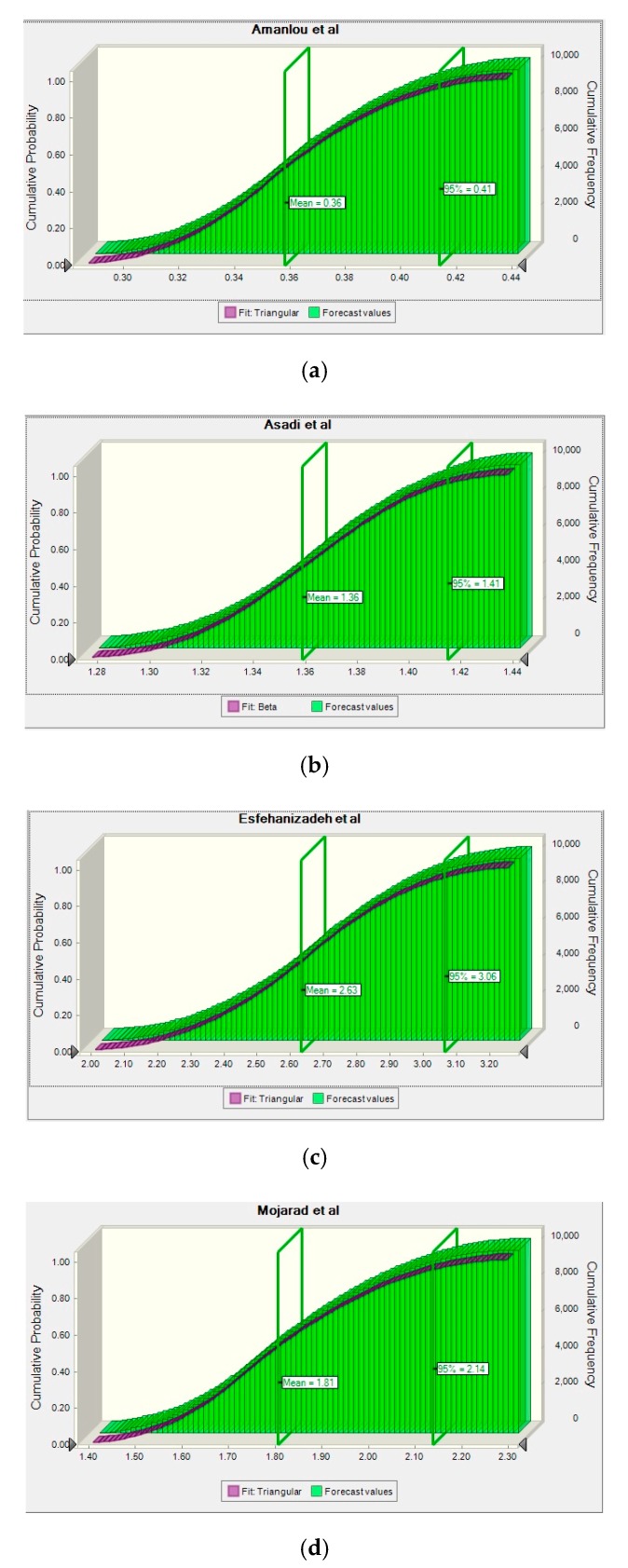

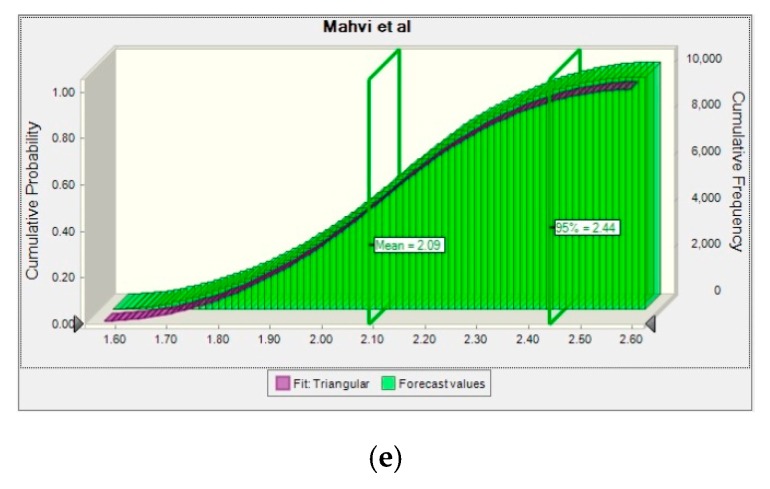
Cumulative probability plot of fluoride concentration (mg/L) in the different studies: (**a**) Amanlou et al [23], (**b**) Asadi et al. [27], (**c**) Esfahanizade et al [26], (**d**) Mojarad et al. [17], (**e**) Mahvi et al. [28].

The consumption of tea as a healthy beverage is popular in Iran and other parts of the world. Tea is prepared from the leaves of *Camellia Sinensis*, a species of flowering plant belonging to the family of Theaceae. Based on various manufacture and processing techniques, the prepared tea can be categorized into black (fermented), green (non-fermented), oolong (partially fermented), puerh (prolong fermented) and white (un-oxidized) [19,37]. Usually, tea is consumed after infusing tea leaves for a few minutes using hot water. Although tea has therapeutic properties, it can be a major source of fluoride exposure and thereby poses risk to the consumers. Thus, it is important to determine the concentration of this element in tea to decrease its harmful health effects. By referring to the literature, all the studies published regarding fluoride in consumed tea in Iran were selected in order to investigate the overall risk to the population associated with fluoride in tea. The levels of fluoride in published studies varied significantly, ranging from 0.13 to 3.27 mg/L. Figure 2 shows the cumulative probability plot of the measured fluoride concentration (mg/L) in these studies. This varying range of fluoride in consumed tea can be attributed to the various types of tea, brewing time, etc.

### 3.2. Non-Carcinogenic Risk Assessment

The health benefits of various types of tea have been investigated in recent years [3]. Nevertheless, the adverse effects of unwanted fluoride from drinking tea on human health is a question that cannot be overlooked while taking into account its healing benefits. Estimation of the chronic daily intake (CDI) of exposure frequency (F) in tea consumers can be effective in assessing the risk involved [20]. The fluoride concentrations derived from five studies were applied to determine the CDI values via the ingestion contact route. The CDI value of exposure of men, women, and children to fluoride in consumed tea is shown in Table 3. The CDI values for tea based on the 95 percent percentile varied from 9.23 × 10^−3^, 6.74 × 10^−2^, 2.84 × 10^−4^, 2.07 × 10^−3^, 1.96 × 10^−5^, 1.43 × 10^−4^ mg/kg/day for men, women, and children, respectively.

The obtained results showed that the health risk in the women and children groups was well within the safe zone (HQ <1) during all the years, which shows that consumed tea does not lead to adverse health effects. However, in 20 percent of cases in the men groups, the HQ was higher than 1, which implies a non-carcinogenic health risk for this group. From the obtained results it can be concluded that the non-carcinogenic risks of fluoride for the three exposed populations were in the order of men > women > children. The results of this study were compared with the results of studies conducted in other parts of the world. In a study conducted by Waugh et al. fluoride concentration in most consumed brands of tea in the Republic of Ireland was investigated. They found that the fluoride concentration in all the tested samples ranged from 1.6 to 6.1 mg/L, with a mean value of 3.3 mg/L. In addition, the majority of inhabitants in the Republic of Ireland were at a high risk of chronic fluoride exposure and related adverse health effects according to established reference levels [38]. Sofuoglu and Kavcar estimated fluoride exposure and associated health risk in black tea in Turkish people. In their study, fifty participants were randomly selected, and non-carcinogenic health risk assessment was evaluated based on the CDI and HQ model. Their results revealed that fluoride levels in black tea were not found to be associated with a considerable health risk [20].

In another study, Malinowska et al. investigated the fluoride content in infusions of commercially available black, green, oolong, puerh, and white teas after 5 min of brewing. They found that the fluoride concentration was 0.32 to 4.54 in black, 0.59 to 1.83 in green, 0.59 to 1.83 in oolong and 0.37 to 0.54 mg/L in puerh [16]. Cai et al. studied fluoride concentration in most consumed tea in China during 2014–2015. Their results showed that the fluoride concentration ranged from 5.0 to 306.0 mg/kg, with an average of 81.7 mg/kg. Moreover, based on statistical analysis by Monte Carlo simulation and the HQ, there was no risk of fluorosis from drinking tea [12].

Peng et al. determined the fluoride concentration in commercial teas in China and estimated the CDI related to consumption of these teas in the period from 2010 to 2013. The six types of tea were selected as green, black, oolong, puerh, white, and reprocessed. Their results showed that fluoride concentration mean was 63.04, 99.74, 52.19, 101.67, 159.78, and 110.54 mg/kg for the mentioned tea types, respectively. Based on the Monte Carlo simulation, the simulated average daily intake of fluoride for green, black, oolong, puerh, white, and reprocessed tea were 0.64, 1.27, 1.91, 1.27, 0.64, and 1.27 mg/day, respectively [39]. The rate of consumption of tea in a different climate is completely variable, however, personal habits and cultural situations also affect daily intake of fluoride [40]. Since tea is one of the potential sources for fluoride intake in humans, identification of brands that contain high levels of fluoride should be considered and, the necessary information should be given to people. The obtained results can be helpful as a benchmark to organizations such as the ministry of health and agriculture. It should be noted that some other sources of fluoride such as drinking water or food and fluoride supplements can also be associated with daily intake of fluoride. Thus, conducting a probabilistic health risk assessment for the studied people could be underestimated since other sources of fluoride have not been considered. Based on the finding, children in the developmental growth stage are considered a sensitive group due to their weak immune systems and high vulnerability.

## 4. Conclusions

As tea is the most popular drink in the world after water, it can be considered an important part of a healthy diet. Many beneficial facets of tea are now scientifically proven, and it could be prudent to encourage its regular consumption as an alternative to other drinks. However, based on an earlier literature survey, strong evidence has been revealed that tea can be a high and detrimental source of fluoride intake in humans. The excess intake of fluoride can lead to health problems as fluoride can cause fluorosis, skeletal fluorosis etc. In Iran, as one of the tea producers in the world, there is a high tendency for tea drinking. The concentration of fluoride in all the studies in the literature ranged from 0.13 to 3.27 mg/L. The obtained HQ values for the age groups of women and children were under the USEPA guideline value of one, showing that there was no probable human health risk in terms of tea consumed via the ingestion pathway. Only in 20 percent of samples in men groups, was HQ higher than one. It should be noted that the assessment of health risk from the exposure of fluoride in tea without considering other sources of fluoride could lead to underestimation. Although, in most cases the level of fluoride found was low, much effort is still needed to minimize further exposure of fluoride to humans.

## Figures and Tables

**Table 1:** General information of the related studies.

Reference	Location	Tea Type	Brands	Sample	Time	Fluoride Concentration (mg/L)			SD
			Type	Number	Year	Mean	Min	Max	
[23]	Tehran-Iran	Tea Bag	15		2008	0.26	0.13	0.44	0.01
[26]	Tehran-Iran	Tea Bag	6	36	2010	2.02	0.7	3.27	1.06
[27]	Qom-Iran	Tea Bag	4		2012	1.28	1.11	1.44	
[28]	Tehran-Iran	Tea Bag	10		2006	1.63	0.53	2.6	0.16
[17]	Hamadan-Iran	Tea Bag	22		2012	1.139	0.48	2.3	

**Table 2:** Input parameters of the risk model.

Parameters	Symbol	Men	Women	Children	Unit
Average contamination concentration	C	-	-	-	mg/L
Intake rate	IR	2	2	1	liter/day
Exposure frequency	EF	365	365	365	day
Exposure duration	ED	40	40	6	a/life time
Body weight	BW	78	65	14.5	kg
Average time	AT	14,600	14,600	2190	days

**Table 3:** Chronic daily intake (CDI, mg/kg/day) and target hazard quotient (THQ) values due to exposure of men, women, and children to fluoride in consumed tea based on 95 percent percentile obtained from Monte Carlo simulations.

Reference	Con. (Max)	CDI			THQ		
		Men	Women	Children	Men	Women	Children
[23]	0.36	9.23 × 10^−3^	2.84 × 10^−4^	1.96 × 10^−5^	0.154	4.73 × 10^−3^	3.26 × 10^−4^
[26]	2.63	6.74 × 10^−2^	2.07 × 10^−3^	1.43 × 10^−4^	1.12	3.46 × 10^−2^	2.39 × 10^−3^
[27]	1.36	3.49 × 10^−2^	1.07 × 10^−3^	7.40 × 10^−5^	0.581	1.79 × 10^−2^	1.23 × 10^−3^
[28]	2.09	5.36 × 10^−2^	1.65 × 10^−3^	1.14 × 10^−4^	0.893	2.75 × 10^−2^	1.90 × 10^−3^
[17]	1.8	4.62 × 10^−2^	1.42 × 10^−3^	9.79 × 10^−5^	0.769	2.37 × 10^−2^	1.63 × 10^−3^
